# Essential amino acids: master regulators of nutrition and environmental footprint?

**DOI:** 10.1038/srep26074

**Published:** 2016-05-25

**Authors:** Paolo Tessari, Anna Lante, Giuliano Mosca

**Affiliations:** 1 Dept. of Medicine, University of Padova, Italy (PT); 2 Dept. of Agronomy, Food, Natural Resources, Animals & Environment (DAFNAE), University of Padova, Italy (AL, GM)

## Abstract

The environmental footprint of animal food production is considered several-fold greater than that of crops cultivation. Therefore, the choice between animal and vegetarian diets may have a relevant environmental impact. In such comparisons however, an often neglected issue is the nutritional value of foods. Previous estimates of nutrients’ environmental footprint had predominantly been based on either food raw weight or caloric content, not in respect to human requirements. Essential amino acids (EAAs) are key parameters in food quality assessment. We re-evaluated here the environmental footprint (expressed both as land use for production and as Green House Gas Emission (GHGE), of some animal and vegetal foods, titrated to provide EAAs amounts in respect to human requirements. Production of high-quality animal proteins, in amounts sufficient to match the Recommended Daily Allowances of all the EAAs, would require a land use and a GHGE approximately equal, greater o smaller (by only ±1-fold), than that necessary to produce vegetal proteins, except for soybeans, that exhibited the smallest footprint. This new analysis downsizes the common concept of a large advantage, in respect to environmental footprint, of crops vs. animal foods production, when human requirements of EAAs are used for reference.

The “environmental footprint” in food production is a key issue in modern times. The steep increase of the world population, requiring more and more food for adequate nutrition, the progressive use of land for the production of animal and vegetal foods, the waste of consistent surfaces that could instead be devoted to agriculture, are all factors potentially compromising the provision of adequate nutrition for humanity in the near future.

The Food and Agriculture Organization (FAO) of the United Nations has recently estimated that ≈850 million people, i.e. ≈15% of the world’s population, are chronically hungry nowadays, and that even more suffer from nutritional inadequacy^1^. About 1-billion face an inadequate protein intake, causing a variety of nutritional deficiencies, impaired growth, poor health etc^2^. Prospectively, ≈70–100% more food than that produced today will be required by 2050^3^. Therefore, a dramatic increase in the demand of land, the need for increased efficiency in the food production system, and/or a reconsideration of dietary habits in the perspective of human requirements, are to be expected in the near future.

Two widely-used parameters employed to quantify the environmental footprint of food production are land use and the Green House Gas Emission (GHGE). Both land use and GHGE depend on production systems (e.g. the yields per surface, the efficiency of the processes), and on lifestyle/tradition/consumption patterns of a given population^4^. Therefore, both food production and consumption habits exert a large impact on both land use and GHGE, approximately to the same order of magnitude^4^.

It is commonly accepted that production of vegetables and grains results in a much lower environmental footprint than that required for meat and other animal foods production^5^. Conversely, a given agricultural surface employed to cultivate vegetables would theoretically nurture more people, than if used for meat, poultry, or dairy foods production.

The key question in such a comparison, however, is that about the concept of “more food”. Food can be quantified as weight, caloric density (kilocalories over weight), nutritional value (the nutrient content in respect to the Recommended Daily Allowances, RDAs), or, more generally, from a “qualitative” standpoint^6^. The choice among either of these parameters has a great impact on the calculation of the relationship(s) between the amount of food produced, and the associated environmental impact. Using calories as reference parameter, it is popularly perceived that the production of bovine meat causes a much greater environmental footprint than that of *isocaloric* amounts of dairy foods, eggs, and even pork meat (see as an example: Kunzing, R. Carnivore’s Dilemma. National Geographic Magazine Nov. issue, 109–135, 2014). Nevertheless, when the environmental impact associated to production of hundreds of foods and beverages, was analysed in respect to both energy density (expressed as ratio between calories and weight), and “nutritional density” (i.e. “the sum of percentage daily values of “n” nutrients, calculated per 100-kcal reference amount”), a different picture emerged, markedly blunting or even abolishing the theoretical advantage, in terms of land use and GHGE, of crops production and vegetal food consumption^7^. Other reports came to nearly similar conclusions^8^,^9^. Although valuable, these as well as other previous investigations addressing the issue of the environmental footprint of food production, did not take into consideration one key factor of food quality, namely the content of essential amino acids (EAA) in the proteins vs. their daily requirements for human beings^10^. The total protein content of the various foods was actually considered, not their nutritional values in terms of EAAs. Although such a concept might indirectly have been included into that of the “nutritional adequacy” of food products, such a method of analysis was never directly employed.

Proteins are major nutritional components, providing both non-essential and essential amino acids. The latter, by definition cannot be synthetized by the body in humans, who therefore depend on nutrition for their provision.

Sources of proteins can be either animal or vegetal foods. Broadly speaking, the nutritive value of vegetal proteins is lower than that of animal ones, because the former have a deficient and/or an unbalanced EAAs content^11^,^12^. Therefore, it could be somewhat more difficult to guarantee the RDA of all the EAAs using only vegetal, rather than animal or mixed vegetal/animal protein feeding^11^,^12^. In other words, an individual would need to eat more vegetal proteins to get the same level of nutrition as that offered by the animal ones. Therefore, since the production of proteins of either source has a relevant and differential environmental footprint, the consumption and/or the design of diets adequate in dietary proteins and EAAs, but from different sources, do retain a major ecologic footprint^13^.

Therefore, the aim of this study was to re-assess the environmental footprint, expressed both as the land surface required for production, and as GHGE, of selected foods of either animal or vegetable sources, in respect to their EAAs content and daily requirements for humans.

## Methods

Following an extensive survey of scientific literature, we retrieved from published reports and databases, the land surface and GHGE estimates for the production of a limited number of “sample”, popular foods, of both animal and vegetal origin.

We retrieved also the data about their edible fractions and amino acid composition. These data were comprehensively analysed to provide estimates of the environmental footprint associated to the production of specific amounts of these sample foods.

### Land use for production of standard amounts of sample food products

The land surface required to produce animal foods is affected by a variety of factors, such as the animals’ species, their nutritional requirements, the nutritive value of the feed employed, the agricultural yield of the feed ingredients, etc. These factors can also vary widely among latitudes, countries, agricultural habits etc^14^. Therefore, an “average” common factor relating the production of sample animal foods to land use may be very difficult to calculate, and it may not be representative of all environmental conditions. Given these limitations however, following an extensive and critical literature analysis, we selected land use data typical of central Europe and applicable also to northern Italy. The same approach was employed for the selected vegetal foods, with the exception of quinoa, that is not yet extensively produced in Europe, and for which we used data mostly typical of southern America.

In general, when the land use data for a given food varied markedly among published reports, we selected those data that were closer to the mean of reports, i.e. we did not consider extreme values.

Land use data were first referred to production of a standard amount (1 Kg, or 1 L for cow milk) of foods (Table 1). When multiple estimates were found, their average value was chosen for the calculations (Table 1, data typed in “bold”). Thus, land use for production of eggs were found in refs. 4, 13, 14, 15, 16; for cow whole milk, in refs. 4, 14, 17, 18, 19, 20; for beef meat, in refs. 4, 14, 15, 21, 22, 23; for pig meat, in refs. 4, 14, 16, 21, 23, 25; for chicken, in refs. 13, 14, 16, 21, 26, 27; for aquaculture fish (sea bass), in refs. 14, 28, 29; for soybeans, in refs. 30, 31, 32, these estimates being rather close to those calculated at our University by one the coauthors of this paper (G.M., personal data); for beans, in (ref. 13, Fig. 9 on page 11) refs. 32, 33; for peas, in refs. 32, 34, 35; for wheat flour, in refs. 4, 13, 35, 36; for maize, in refs. 37, 38; for rice, in ref. 39; for potato, in refs. 26, 31; for cauliflowers, in ref. 40; and, finally, for quinoa, in refs. 32, 41, 42, 43, 44, 45, 46.

### Edible parts of the foods

Starting from the raw weight of the foods, an accurate estimate of their edible fraction is required, in order to correctly associate the food fraction, viable for effective nutrition, to land use. Therefore, when this fraction was different from 100%, corrections had to be introduced. These calculations were again carried out after an extensive literature survey (Table 1), with the exception of eggs, for which we used a standard, common egg weight of 60 g, with an edible part of 55 g (5 g being represented by the shell). Therefore, 1 kg of edible egg would correspond to 18,18 eggs. We also assumed that albumen accounts for 40 g, and yolk for 15 g, of the 55 g of the egg edible part^47^. The calculation of egg amino acid composition and content (see below) was carried out separately for these two fractions and then combined. For pig meat, the edible part was assumed to be 79% of the raw meat weight^47^; for chicken, 58% of the entire animal, a figure resulting from a live animal-to-carcass recovery of 72.5%, (the average of data from Elferink^21^ and Njidam^14^), further corrected for a carcass-to-meat recovery of 80%, and for an edible fraction of 98%^47^; for sea bass (as fillets), 40% of fish weight, on turn resulting from a 90% “dressing percentage”, from which fillet yield is 44.1%^48^; for soybeans, 88,1%^47^; for beans, 52%^47^; for peas (as fresh product), 31%^47^; for wheat flour, 75%, i.e. the average flour yield from wheat^49^; for maize flour, 80%^50^; for rice, 62%^51^; for potato, 80%, i.e. the mean of data from^26^,^31^; for cauliflowers, 58%, i.e. the average between data from^40^,^47^; for quinoa, 90.6%^52^.

### Green-House-Gas-Effects (GHGE)

The GHGE data (expressed as total CO_2_ per Kg of each food product) were retrieved from published reports (refs. 13, 14, 29, 33, 37, 45, 46, 47, 53, 54, 55), usually through the Life Cycle Assessment (LCA) methodology, and are summarized in Table 2. When either ranges or multiple data were available, we used the gross mean of these estimates. As regards chicken, we used mixed GHGE values of chicken and poultry^13^,^14^,^29^, since there are limited data referred just to chicken. As regards fish, we used the average of data from common aquaculture productions, not just for the sample fish we chose, i.e. sea bass, because there are no such estimates.

### Amino acid composition data

The amino acid composition of most foods were derived from the database of the Italian National Institute for Research in Food and Nutrition (INRAN)^47^. Exceptions regarded quinoa, for which we used data reported in ref. 57 (using the mean between the Q9 and Q11 fractions); soybeans, for which we used the average amino acid compositions resulting from pooling the data of refs. 58, 59, 60; potato and cauliflowers^61^. For beef meat amino acid composition, we used the INRAN data^47^ referred to the “*rump*” cut of adult animal, i.e. a medium-to-high quality cut, that has an amino acid composition very close to that of other good quality cuts. For pig, we used the amino acid composition of the pork loin of a medium-size animal. The RDA values for the EAAs, referred to a 70-kg man, were those of the WHO/FAO/UNU 2002 report^10^.

### Calculation of amino acid content in the foods

We calculated the amino acid content of three different amounts of each food product, after correction for the edible fractions.

**Figure 1:**
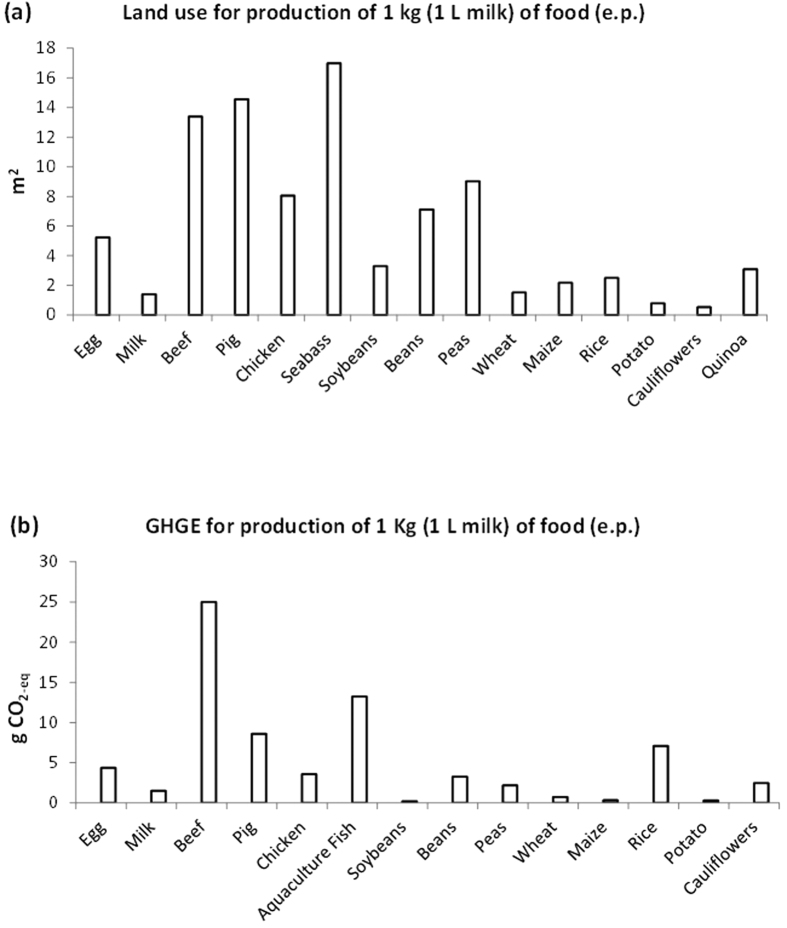
(**a**) Estimated land use (surface, in square meters, m^2^) necessary to produce a standard amount of each food product. Data are referred to 1 Kg (or to 1 L of milk) of the edible part of the foods. (1 Kg egg correspond to 18.18 eggs). See text for references. (**b**) Estimated Green House Gas Emission (GHGE, in Kg CO_2-eq_ kg^−1^), necessary to produce a standard amount of each food product. Data are referred to 1 Kg (or to 1 L of milk) of the edible part of the foods. (1 Kg egg correspond to 18.18 eggs). See text for references.

First, we calculated the EAA composition of 100 g (or 100 mL for cow milk) of the foods, i.e. a standard value corresponding to one tenth of the value of 1 kg (or 1 L for cow milk) as reported in Fig. 1. This was defined as the [A] amount (Tables 3, 4, 5, [Table t3], [Table t4], [Table t4], [Table t5]), and it was arbitrary chosen because it most closely approached the RDA of the total as well as of each individual EAA, provided by high quality protein-containing foods.

Second, we calculated a [B] food amount, that would provide a total of 13 gr of EAAs, i.e. approximately the same total amount of EAAs as that recommended for a reference 70-kg man^10^. Such an amount however, despite matching the “total” EAA requirements, in most foods was nevertheless deficient in some EAAs (see also supplementary Tables 1 and 2, of the additional data).

Finally, we calculated food amounts, defined as [C]), that would provide and match the RDA of each individual EAA, i.e. up-graded to provide the RDA requirements of the limiting EAA. Obviously, this amount resulted in the excess of all the other, non-limiting EAAs (as shown in Supplementary Tables 3 and 4 of the additional data).

Using the three, above described, quantities of each food product (expressed as edible parts), the corresponding values of land use (in square meters, m^2^) and the GHGE (as total CO_2-equivalent_ released per Kg of product), were derived.

## Results

### Essential Amino Acid content in the selected food products

The content/composition of essential amino acids of the food products, expressed per 100 g of edible part (the [A] quantity), are reported in Table 3 (animal foods) and Table 4 (vegetal foods). Among the former, lean bovine meat (“beef”) more closely approached the total as well as the individual RDA of all EAAs, although there were still some minor deficiencies. Among the latter, soybeans were the closest to the EAA RDAs, and they actually provided an excess of most EAAs (both as total and also individually), whereas most other vegetal foods were clearly deficient in many of them.

When the food amounts were recalculated to provide ~13 g of *total* EAAs [B], all food quantities, with the exception of soybeans, had to be increased to a variable extent, above the [A] value of 100 g (or 100 mL for milk) (Table 5, [B] set of data). Only the amount of soybeans had to be decreased. However, despite such an adjustment, many EAAs were still below their RDA in most foods (see Supplementary Tables 1 and 2 of additional data, for individual amino acid composition and content of the different foods). For instance, while beef meat was only slightly deficient in leucine, eggs were relatively more deficient in leucine and histidine, milk in lysine, histidine and [cysteine + methionine], beans and soybeans in lysine and [cysteine + methionine], wheat in lysine, and peas in histidine, [cysteine + methionine] and leucine.

Finally, when the amount of each food was titrated to provide the RDA of the limiting EAA (the [C] quantities), greater amounts of each food were obviously necessary (Table 5, [C] set of data). This lead also to an excess of the other EAAs in respect to RDA The lowest increments were however observed for animal-derived foods, the highest for vegetal foods (with the exception of soybeans).

### Land use data

The land use data referred to production of 1 Kg (or 1 L for milk) of each food, derived from the data reported in Table 1, and corrected for the edible part of each food, are shown in Fig. 1a. The production of lean beef and pork meat, and of sea bass, required the greatest land surface, followed by egg, chicken, pea, beans, most vegetal foods showing the lowest values.

**Figure 2:**
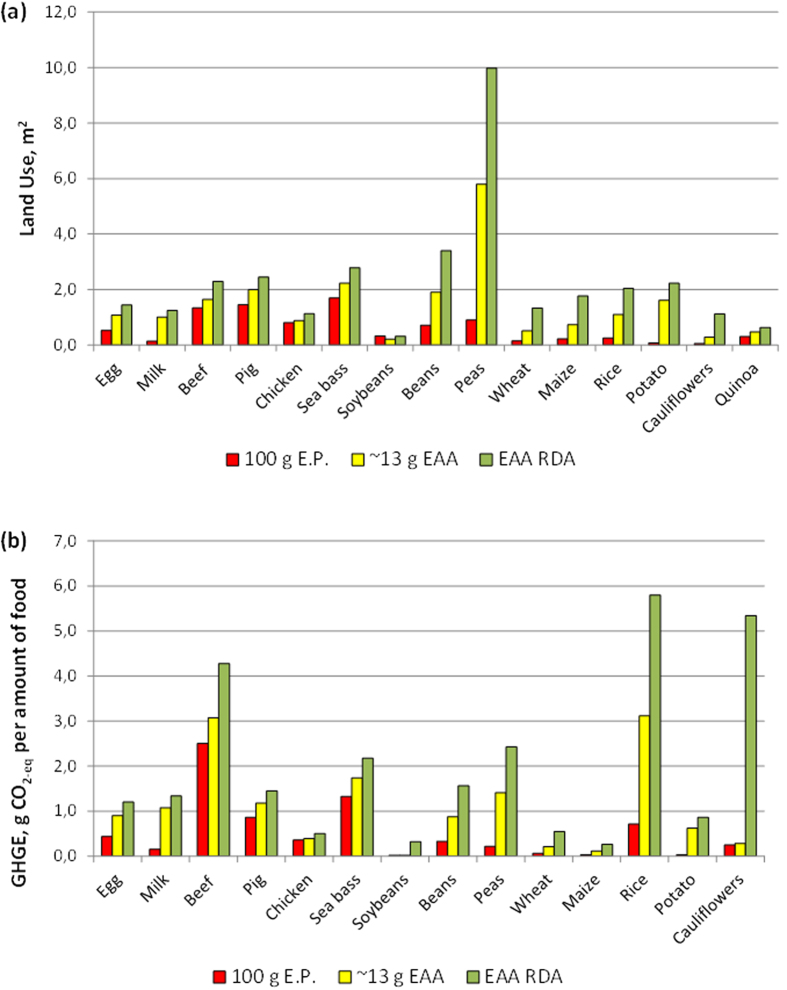
(**a**) Estimated land surface (in square meters, m^2^) necessary to produce either 100 g (mL for milk) of each standard food product [A] (left bars in each triplet); an amount sufficient to provide 13 g of total essential amino acids (EAA) (middle bars) [B], or the RDA of all EAA, i.e. matching the RDA of the limiting amino acid (right bars) ([C]. Data are referred to edible amounts of each food. (**b**) Estimated Green House Gas Emission (GHGE, in Kg CO_2-eq_), necessary to produce either 100 g (mL for milk) of each standard food product [A] (left bars in each triplet); an amount sufficient to provide 13 g of total essential amino acids (EAA) (middle bars) [B], or the RDA of all EAA, i.e. matching the RDA of the limiting amino acid (right bars) [C]. Data are referred to edible amounts of each food.

However, when land use was recalculated with respect to either the production of 15 g of *total* EAAs from each food product ([B] amount), or to ensure the RDA of *all* EAAs [C], and compared to those of the [A] amount, the results were quite different (Fig. 2a).

**Figure 3:**
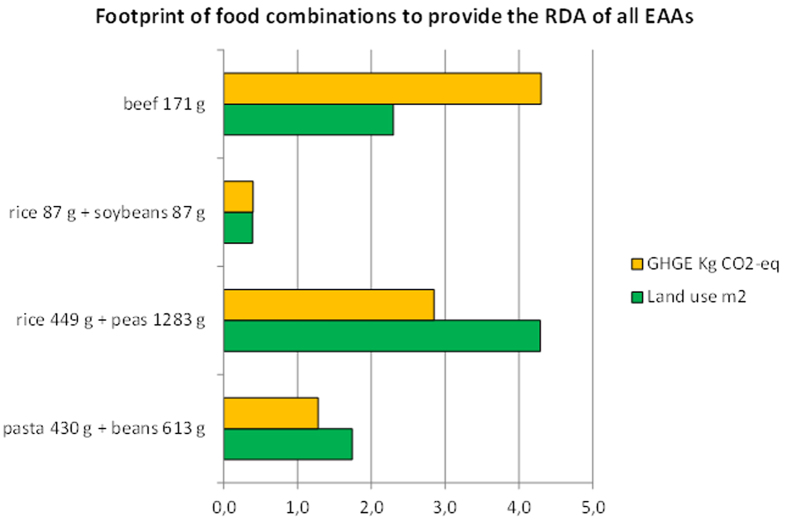
Environmental footprint, expressed either as GHGE (in kg CO_2-eq_, orange bars) or as land use (in m^2^, green bars) of three sample combinations of cereals and legumes as compared to beef. The amount of each food combination, as well as of beef, is calculated to provide the RDA of all the EAAs. The resulting raw weight of each food is reported in the y axis. The proportions between the two foods in each cereal/legumes combination reflected common practice and tradition (i.e. 0.7/1 grams for pasta/beans; 0.35/1 grams for rice and peas; 1/1 grams for rice and soybeans). Dried pasta was assumed to contain 88% wheat. The calculations were performed using the edible parts of each food, and back-calculated to yield the raw weight of each food.

While land use for beef and soybeans production was only minimally affected using either amount, there were marked differences among the three chosen food amounts as regards beans, peas, wheat, maize and, to a lesser extent, rice and cauliflowers. The estimated land use to produce food amounts satisfying the RDA of each EAA actually became approximately equal to than that required for beef production for most foods (Fig. 3), with the exceptions of egg, milk, chicken, quinoa and soybeans, the latter still requiring ≈85% less land than that of beef meat. Notably, land use was the greatest for peas and beans production.

### GHGE data

The GHGE data referred to production of 1 Kg (or 1 L for milk) of each food, derived from the data reported in Table 2, and corrected for the edible part of each food, are shown in Fig. 1b. The production of lean beef meat and of fish (from aquaculture) required the greatest land surface. In contrast, the lowest figures were associated to milk (however on a *pro-liter* basis), egg, and, in general, to vegetal foods, with exceptions for beans and rice (Fig. 1b).

When referred to production of either approximately 13 g of *total* EAAs (Table 5, [B] dataset), or to ensure the RDA of *all* EAAs (Table 5, [C] dataset), also GHGE data were quite different from those associated to the [A] amounts (Fig. 2b).

While the GHGE for animal foods as well as for soybeans was only minimally affected using either amount, differences were greater among the [A], [B] and [C] amounts of most vegetal foods (beans, peas, wheat, rice and cauliflowers). The estimated GHGE for food amounts satisfying the RDA of each EAA actually became approximately equal to that of beef and sea bass, for peas and rice, ~40% greater for cauliflowers, while the gap between beef or fish, and beans, peas, wheat and potato was reduced. Only soybeans still required ≈90% less land than beef meat.

### Food combinations

We also calculated the required land and the GHGE figures relative to sample food combinations. We arbitrarily selected three combinations (“plates”) typical of some peoples and/or cultures, i.e. that between cereals (either rice or wheat) and legumes (peas, beans or soybeans). The proportions chosen in each plate between the two contributing foods reflected common practice and tradition. These data are reported on Fig. 3, and compared to those of beef. Only the combination including soy beans showed an environmental footprint markedly lower than that of beef. Note also the great amounts of rice and peas, as well as of pasta and beans, required to satisfy the EAA RDAs.

## Discussion

In this study we estimated the environmental footprint, expressed both as land use and as GHGE, associated to production of standard amounts of selected, reference foods, in respect to the requirements of essential amino acids for humans. The main conclusion of the study is that, under this perspective, the theoretical advantage of producing vegetal rather than animal proteins, is either markedly blunted, abolished or even reverted, with the notable exceptions of soybeans (still requiring ≈85% less land and producing ≈90% less GHGE, than those associated to beef meat). Also the production of other vegetal products (wheat, maize, cauliflowers and quinoa, Fig. 2a) required less land, and resulted in a lower GHGE (maize, beans, wheat and potato, Fig. 2b) than beef. However, large amounts of vegetables were required to comply with the RDA of all the EAA (with the exception of soybeans), as compared to animal proteins (Table 4).

Our calculations were targeted to the content of the essential amino acids in the foods. Essential amino acids are key components of diet. The RDA of each EAA had been established in extensive studies and reported by international organizations^10^. In this study, we adopted the EAA RDA values from north American studies, because similar data from European studies are not available. Therefore, we had to assume that the EAA RDAs are not different between American and European populations.

The EAAs by definition are indispensable substrates, since they cannot be *de-novo* synthesized by the body, and their provision depends on the intake of protein-containing foods. The EAAs are to be ingested in specific amounts and appropriate proportions daily, to ensure a physiological body protein synthesis, a normal growth in babies and adolescents, the maintenance of the body protein pool and recovery from catabolic states. In other words, they are key substrates to either preserve or regain body protein mass^11^,^12^. The adequate provision of EAAs depends therefore on the quality of the dietary proteins, and the EAAs content should be taken as one of the reference parameters, when defining the nutritional quality of a given food.

Previous investigators used a variety of approaches and parameters to estimate the “nutritional quality” of foods, also called “nutrient profile”, that were then implemented in different models^6^,^8^. The list of previously-used parameters, variably combined, include: 1) the nutrient content per 100 g of edible portion; 2) the daily recommended values for nutrients, with proteins considered as a whole; 3) the number of nutrients contained in a specific food; 4) the so-called “nutrient adequacy scores”; 5) the “nutrient density score”; 6) the energy density of foods (kcal/g); 7) the “limited nutrient score”; 8) the “maximum recommended values”, or, simply: 9) the caloric content^6^,^8^. By employing either of these models, estimates of the environmental footprint associated to nutrient consumption had been calculated^6^,^8^.

The use of either of these terms and concepts doesn’t have only a semantic relevance, but it essential when transferring the nutritional parameters of food quantity and quality, to the environmental footprint associated to their production. For instance, if one considers just food weight, weight itself doesn’t obviously guarantee an adequate content of all the required nutrients. The same concepts applies to the energy content of food. As a matter of fact, food calories can be associated to nutrients with markedly different nutritional values in respect to daily recommended allowances. Therefore, not all foods, despite a similar caloric content, have the same “nutritional value”, if based on most specific and appropriate parameters, such as that of their EAAs content. Our way of analysis unveils a much more complex relationship between the environmental footprint of foods and human requirements. As a matter of fact, previous studies reported a several-fold greater environmental footprint for cattle breeding and meat production, than that associated to production of other types of animal or vegetable food products^5^,^7^.

In this study, as indexes of the environmental footprint in food production, we used both land use to produce a given amount of food, and GHGE. These parameters are two of the most relevant ones in the evaluation of the environmental footprint of the food production chain, in addition to water use, animals’ waste, production of animal feeds and fertilizers, etc. that on turn may (partially) be included in the GHGE itself 6. Therefore, our choice is only indicative, although it considers two of the most relevant factors^6^. Furthermore, both land use and GHGE may markedly vary among climates, traditions, agricultural techniques, and they are *per se* difficult to be accurately determined. Therefore, our conclusions should be taken with caution.

Our data may provide a new approach, although schematic and/or theoretical, to determine, perhaps more accurately, the environmental footprint associated to the production of both animal and vegetal foods.

In a recent report^8^, hundreds of foods and beverages were analyzed in respect to both their environmental footprint and their “nutritional value”. The latter was estimated using a complex score system, based on the ratio of nutrient content (expressed as percent of daily requirements) to calories, as well as on a “nutrient density score”. A view quite different from that commonly perceived emerged, markedly blunting the theoretical advantage, at least in terms of the GHGE of vegetal production^8^. The same concept was highlighted in other recent publications^10^,^13^. These studies therefore are in agreement with our conclusions, however based on a different methodology.

Although combinations of foods can mutually compensate for individual EAA deficiencies, on the basis of our calculations there were not marked advantages when cereals and legumes were combined (Fig. 3), with the exception of soybeans (here combined with rice), that exhibited the lowest environmental footprint also associated to limited amounts of each food. Notably, combinations of wheat and beans, and rice and peas, resulted also in large amounts of each of these foods to be assumed, to comply with the EAAs RDA (Fig. 3).

The EAAs are not the only essential substrates for human nutrition. Nevertheless, their provision is usually more costly and less immediately feasible, that that of other “essential” nutrients, such as water, vitamins, essential fatty acids, salts and minerals. While the latter can be produced and/or recovered from various sources in nature, the production of the EAAs depends either on that of proteins, or on costly extraction and manufacturing processes. In this respect, the addition to foods of selected EAAs to compensate for specific deficiencies, may theoretically be another valid, cost-efficient procedure, with the aim to increase the nutritional value of a variety of food products and to simultaneously decrease land usage for food production.

Another important theoretical issue is that of the appropriateness and the health-related quality of foods. From the data reported on supplementary Table 4, it is evident that, should single vegetal food products provide the entire RDAs of each individual EAA, the intake of the non-limiting essential amino acids, as well as of the non-essential ones, would be variably, often markedly, increased. For some foods, such an excess would also be associated to a greater caloric intake, because of the co-ingestion of either starch or other high caloric substrates there contained. Consequently, all these beyond-requirement intakes will determine a marked excess of substrate oxidation, energy expenditure, reactive oxygen species production, fat deposition, gluconeogenesis, etc. Therefore, the medium- as well as long-term effects of such a metabolic “overflow” need to be accurately evaluated.

The absorption of proteins contained in vegetal foods (including grains and legumes) may be limited and/or somehow impaired because of the presence of fibers as well as anti-nutritional compounds, particularly in soy^30^. These characteristics should be taken into consideration as regards the overall nutritive value of foods.

An estimate of EU options regarding agricultural land use for the yrs 2000–2030, did not show a clear advantage of the switch to the so-called “healthier diets” (i.e. with less environmental impact) in substitution for either meat or other animal products^29^, therefore in a broad agreement with our findings.

Finally, we would not suggest that either meat or other animal-derived proteins should be preferred and/or recommended over that of vegetal ones. The choice between mixed vs. vegetarian (an/or vegan) diets retains many and important cultural, environmental, economic, even psychological implications and connections, that definite statements cannot be made. Surely, a (mild) restriction of meat proteins could be safe for both human health and environmental sustainability^9^, given also the common excess of dietary proteins in western diets and recent warnings about the possible association between red meat consumption (particularly processed meat) and global mortality^62^. The main object of this study was simply to provide direct, theoretical data, on the environmental impact of the production of some sample foods in respect to human EAA requirements.

In conclusion, our data show that the concept of the “environmental footprint” associated to the production of animal vs. vegetal protein-containing food products, needs to be re-evaluated on the basis of the content of essential amino acids in foods. The production of protein-containing animal foods would retain a (much) lower environmental impact than that previously estimated, approximately lying within the range of that of most foods of vegetal origin, because of the higher quality of animal proteins. These considerations might be useful in the political planning of the food production system, aiming at providing sufficient food for humans in the near future.

## Additional Information

**How to cite this article**: Tessari, P. *et al*. Essential amino acids: master regulators of nutrition and environmental footprint? *Sci. Rep.* **6**, 26074; doi: 10.1038/srep26074 (2016).

## Supplementary Material

Supplementary Information

## Figures and Tables

**Table 1:**
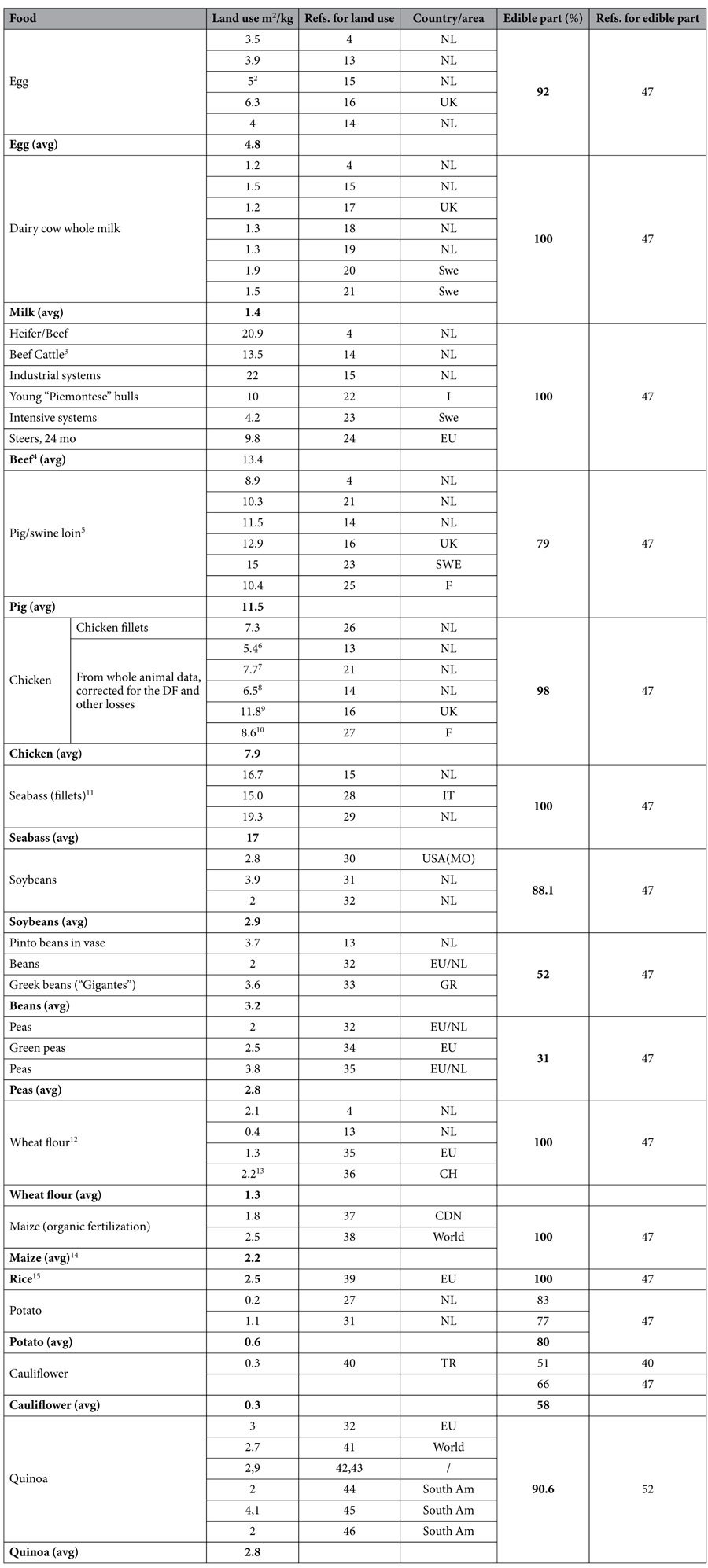
Land use (in m^2^) for the production of standard amounts of foods, and their edible part (%) with reference to literature data.

Data are expressed per 1 Kg (1 L for milk) of either raw food weight, or after correction for the “dressing factor” (DF) or other losses^1^. Original land use data in tons/ha (hectare) were converted to m^2^/kg. The average (avg) value was calculated from multiple references, as indicated.

^1^The “dressing factor” (DF) is the consumable fraction of an animal, after removal of non-consumable parts following the slaughter process.

^2^Average value from ref. 16.

^3^Value for maize feeding.

^4^Based on industrial/intensive systems, and corrected by the authors for the “dressing factors”.

^5^Corrected for a final DF composed by carcass yield and edible meat yield^14^.

^6^Using intensive production systems.

^7^Including/calculated from: land required for feed ingredients production, the feed-to-gain ratio of the animal, the nutritive value of feed ingredient, the dressing factor of the animal, the amount of feed ingredient consumed, the total nutritive value of all the feed ingredients consumed, and the share of waste-streams in broiler feed^21^.

^8^Data expressed for product weight.

^9^Data reported for dead animals, corrected for a final dressing factor of 0.58 (see methods for details).

^10^Data reported for live animals, corrected for a final dressing factor of 0.58 (see methods for details).

^11^Corrected for a final dressing factor of 0.40 (ref. 62).

^12^Corrected for a 0.75 recovery of flour from wheat (ref. 49).

^13^Assuming an average fertilization intensity.

^14^Corrected for a 0.80 recovery of flour from maize (ref. 48).

^15^Corrected for a rice plant yield of 0.62 (ref. 51).

**Table 2:** Green-House Gas Effects (GHGE), expressed as total CO_2_ per Kg of product, from LCA studies.

**Food**	***Raw*** **kg CO**_**2**_**−eq kg**^**−1**^	**Recov. factor**	**Corr. for e.p. kg CO**_**2**_**−eq kg**^**−1**^	**Region**	**Refs.**
**Egg** *(range 2–6)*	**4**	0.92	**4.3**	UE, World	14
**Milk**	**1**–**2**	1	**1.5**	UE, World	14
Beef & veal^1^ *(mixed/industrial systems) (range 9–42)*	26		**26**	NL, World	14, 29
Beef^1^*(range 8–16)^2^*	12		**12**	NL	13
Beef^1^ *(semi-intensive?)*^2^	38		**38**	IRE	13
**Beef (avg)**	**25**	1	**25**		
Pork	6.1			SWE	53
Pig meat^1^ *(range 4–11)*	7.5			UE, World	14, 29
**Pork/Pig (avg)**	**6.8**	0.79	**8.6**		
Poultry^1^ *(range 2–6)*	4			UE, World	14, 29
Chicken^2^	3			NL	13
**Chicken/Poultry (avg)**	**3.5**	0.98	**3.6**		
Fresh fish (aquaculture) *(mean of pangasus* = *4.7, and salmon* = *3.7)*	4.21			NL	54
Seafish (aquaculture) (*range: 3–15*)	9			UE	14, 29
Fish^1^,^2^ *(mean of salmon* = *~2 and cod* = *~3.4)*	2.7			NL	13
**Fish (avg)**	**5.3**	0.40	**13.3**		
**Soybeans** (*organic culture*)	**0.19**	0.88	**0.22**	CAN	37
**Beans**^2^	**1.7**	0.52	**3.27**	NL	13
**Peas**	**0.68**	0.31	**2.18**	SWE, EU	33, 53
Wheat flour *(in Italian pasta, 89% w/w)*	**0.85**	1	0.85	IT	55
Wheat *(organic culture)*	**0.29**	0.75	0.39	CAN	37
**Wheat (avg)**			**0.62**		
**Corn** *(organic culture)*	**0.26**	0.80	**0.32**	CAN	37
Rice (*basmati*)	2.31			UK	55
Rice	6.4			SWE	53
**Rice (avg)**	**4.4**	0.62			
Potato	0,17			SWE	53
Potato	0,31			UK,	55
**Potato (avg)**	**0.24**	0.80	**0.30**		53, 55
**Cauliflower**	**1.43^3^**	0.58^4^	**2.46**	NL	40, 47, 56

The reported “raw” values have been corrected (when necessary, i.e. when not originally reported by authors), for the edible part (e.p.), using individual recovery factors resulting from a combination of the “dressing” factors with other losses. Average values (avg) were calculated from the reported ranges. See also the Method section and Table 1 for further references.

^1^As product at supermarket/retail level.

^2^Approximate value derived from Figs 2 of ref. 13.

^3^This is an indirect calculation of GHGE of cauliflowers as Kg CO_2-eq_/Kg of product, derived from the production of total CO_2_ equivalent of ref. 56, under the assumption the cauliflower GHGE is 84% that of beans (taken as reference) both as Kg CO_2-eq_/Kg of product and as production of CO_2_ equivalent.

^4^Calculated as the average of data from refs. 40 and 47.

**Table 3 t3:** Recommended daily allowances (RDA, for a 70-kg man)^1^ and their composition in essential amino acids (EAAs), of sample animal foods.

***RDA^1^***		**Egg**^2^ **100** **g**	**Milk 100** **ml**	**Beef 100** **g**	**Pig 100** **g**	**Chicken 100** **g**	**Sea bass 100** **g**
	Protein content (g)	12, 1	3, 3	22	20, 7	23, 3	21, 3
*Essential amino acids*							
2100	Lysine	1001	272	2002	1737	2246	2021
700	Histidine	322	93	849	647	937	552
1050	Threonine	674	164	898	919	1160	967
1050	Cysteine + Methionine	740	118	871	780	974	897
1820	Valine	896	233	1063	1243	1384	1044
1400	Isoleucine	741	192	950	1080	1153	914
2730	Leucine	748	355	1892	1624	1955	1655
1750	Phenylalanine + Tyrosine	1247	318	1677	1166	1776	1531
280	Tryptophan	228	50	246	183	273	249
**12880**	**Total EAAs (mg)**	**6597**	**1795**	**10448**	**9379**	**11858**	**9830**

Data are reported for 100 g (or to 100 ml, for milk) of edible parts of the foods ([A] quantities) (see Table 1 for data and references). The protein content and the amino acid composition of the foods are taken from published tables of INRAN (the Italian National Institute for Research in Foods and Nutrition)^47^. The sum of cysteine and methionine, and phenylalanine and tyrosine are reported. Histidine is also included, but it is a conditionally essential amino acid.

RDA: Recommended daily allowances, referred to a 70-kg man, see ref. 10.

^2^100 g of edible part of an egg corresponds to 1, 8 eggs.

**Table 4 t4:** Recommended daily allowances (RDA, for a 70-kg man) and their composition in essential amino acids (EAAs), of sample vegetal foods.

***RDA***		**Soybeans 100** **g**	**Beans 100** **g**	**Peas 100** **g**	**Wheat 100** **g**	**Maize 100** **g**	**Rice 100** **g**	**Potato 100** **g**	**Cauliflower 100** **g**	**Quinoa 100** **g**
	Prot. content (g)	38.9	10.2	5.5	11	8.7	6.7	2.1 g	3.2 g	19, 6
*Essential amino acids*										
2100	Lysine	3047	714	348	239	258	257	92	120	1025
700	Histidine	1170	303	85	228	251	165	28	37	478
1050	Threonine	1843	428	310	310	334	246	59	74	849
1050	Cyst + Meth	1183	238	95	454	307	257	51	63	565
1820	Valine	2176	616	226	452	472	438	99	104	961
1400	Isoleucine	2222	556	201	403	350	306	68	73	808
2730	Leucine	3689	885	342	741	1028	590	96	126	1399
1750	Phe + Tyr	3970	963	345	855	761	588	132	129	1542
280	Tryptophan^1^	618	113	54	116	61	84	/	/	726
**12880**	**Total EAAs (mg)**	**19918**	**4816**	**2006**	**3798**	**3822**	**2931**	**624**	**726**	**8353**

Data are reported for 100 g of edible parts of the foods ([A] quantities) (see text and Table 1 for data and references). The protein content and amino acid composition of the foods are taken from published tables of INRAN (the Italian National Institute for Research in Foods and Nutrition). The sum of cysteine and methionine, and phenylalanine and tyrosine are reported. Histidine is also included, but it is a conditionally essential amino acid.

Cyst + Meth: Cysteine + Methionine. Phe + Tyr: Phenylalanine + Tyrosine.

^1^Tryptophan concentrations in potato and cauliflowers are not reported in ref. 61

**Table 5 t5:** Amounts of sample foods required to provide [B] a total amount of EAAs equal to the recommended daily sum of total EAAs (i.e., ~12.9 g) or [C] the RDA for each individual EAA.

	**Egg**	**Milk**	**Beef**	**Pig**	**Chicken**	**Sea bass**	**Soybeans**	**Beans**	**Peas**	**Wheat**	**Maize**	**Rice**	**Potato**	**Cauliflower**	**Quinoa**
B	206^1^	718	123	137	109	131	65	267	642	339	337	439	2063	1775	154
C	295^2^	890	171	168	140	174	89	478	1105	879	814	817	2856	2169	205

Recommended daily allowances (RDA, referred to a 70-kg man) (see Table 2 for references). Data are expressed in grams (g) of edible parts, with the exception of milk (ml).

^1^Corresponding to 3.74 eggs.

^2^Corresponding to 5 eggs.
