# Supplementary material for: Essential amino acids: master regulators of nutrition and environmental footprint?
Source: Sci Rep. 2016 May 25;6:26074. doi: 10.1038/srep26074 (PMC4897092; doi:10.1038/srep26074)
Supplement: Supplementary Information [file srep26074-s1.doc]

Supplementary materials of the manuscript:

Essential amino acids: master regulators of nutrition and environmental footprint?

**Authors:**

Paolo Tessari, Anna Lante, Giuliano Mosca.

Dept. of Medicine, University of Padova, Italy (PT)

Dept. of Agronomy, Food, Natural Resources, Animals & Environment (DAFNAE), University of Padova, Italy (AL, GM)

**Supplementary Table 1**: Amounts of sample animal foods required to provide a total amount of essential amino acids (EAAs) (≈12.9 g) corresponding to the total daily recommended intake of EAAs for a 70-Kg man (“B” quantity, see methods in the text for details). Such an amount is not tailored to the individual requirements of each essential amino acid, but is expressed as their undifferentiated total requirement. Food amounts are for edible part (See Table 1 for further details).

| **AA** | *RDA[[1]](#footnote-2)* | Egg[[2]](#footnote-3)  206 g | Milk  718 ml | Beef  123 g | Pig  139 g | Chicken  109 g | Sea bass 131 g |
| --- | --- | --- | --- | --- | --- | --- | --- |
| *Essential amino acids (mg)* |  |  |  |  |  |  |  |
| Lysine | 2100 | 1786 | 1952 | 2468 | 2385 | 2440 | 2648 |
| Histidine | 700 | 573 | 667 | 1047 | 889 | 1018 | 723 |
| Threonine | 1050 | 1212 | 1177 | 1107 | 1262 | 1260 | 1267 |
| Cysteine+Methionine | 1050 | 1519 | 847 | 1074 | 1071 | 1058 | 1175 |
| Valine | 1820 | 1652 | 1672 | 1310 | 1707 | 1503 | 1368 |
| Isoleucine | 1400 | 1328 | 1378 | 1171 | 1483 | 1252 | 1198 |
| Leucine | 2730 | 2046 | 2547 | 2332 | 2230 | 2123 | 2169 |
| Phenylalanine+Tyrosine | 1750 | 2376 | 2282 | 2067 | 1601 | 1929 | 2006 |
| Tryptophan | 280 | 389 | 359 | 303 | 251 | 297 | 326 |
| **Total EAAs** | **12880** | **12880** | **12880** | **12880** | **12880** | **12880** | **12880** |

**Supplementary Table 2**: Amounts of sample vegetal foods required to provide a total amount of essential amino acids (EAAs) (≈12.9 g) corresponding to the total daily recommended intake of EAAs for a 70-Kg man (“B” quantity, see methods in the text for details). Such an amount is not tailored to the individual requirements of each essential amino acid, but is expressed as their undifferentiated total requirement. Food amounts are referred to edible part (See Supplementary Table 1 for further specifications).

| *RDA* |  | Soybeans 65 g | Beans 267 g | Peas 642 g | Wheat 339 g | Maize 337 g | Rice 439 g | Potato 2063 g | Cauliflower 1775 g | Quinoa 154 g |
| --- | --- | --- | --- | --- | --- | --- | --- | --- | --- | --- |
| *Essential amino acids (mg)* | |  |  |  |  |  |  |  |  |  |
| 2100 | Lysine | 1970 | 1910 | 2234 | 811 | 869 | 1129 | 1900 | 2130 | 1581 |
| 700 | Histidine | 757 | 810 | 546 | 773 | 846 | 725 | 571 | 657 | 737 |
| 1050 | Threonine | 1192 | 1145 | 1990 | 1051 | 1126 | 1081 | 1215 | 1308 | 1309 |
| 1050 | Cyst+Meth | 765 | 637 | 610 | 1540 | 1035 | 1129 | 1052 | 1112 | 871 |
| 1820 | Valine | 1407 | 1647 | 1451 | 1533 | 1591 | 1925 | 2042 | 1849 | 1482 |
| 1400 | Isoleucine | 1437 | 1487 | 1291 | 1367 | 1179 | 1345 | 1402 | 1301 | 1245 |
| 2730 | Leucine | 2385 | 2367 | 2196 | 2513 | 3464 | 2593 | 1972 | 2234 | 2158 |
| 1750 | Phe+Tyr | 2567 | 2575 | 2215 | 2900 | 2565 | 2584 | 2725 | 2289 | 2377 |
| 280 | Tryptophan^1^ | 399 | 302 | 347 | 393 | 206 | 369 | / | / | 1120 |
| **12880** | **Total EAAs** | **12880** | **12880** | **12880** | **12880** | **12880** | **12880** | **12880** | **12880** | **12880** |

^1^  Tryptophan concentration in potato and cauliflowers are not reported in ref . 61. Therefore, the data were calculated with respect to the other limiting essential amino acid. Cyst+Meth: Cysteine+Methionine. Phe+Tyr: Phenylalanine+Tyrosine.

**Supplementary Table 3**: Amounts of sample animal foods required to provide a total amount of essential amino acids (EAAs) corresponding to the daily recommended intake of each EAA for a 70-Kg man (“C” quantity, see methods in the text for details). Such an amount is thus tailored to the individual requirements of each essential amino acid. All the reported amounts of foods are referred to edible part (See Supplementary Table 1 for further specifications).

| **AA** | *RDA* | Egg[[3]](#footnote-4)  275 g | Milk  890 ml | Beef  171 g | Pig  168 g | Chicken  140 g | Sea bass 174 g |
| --- | --- | --- | --- | --- | --- | --- | --- |
| *Essential amino acids (mg)* |  |  |  |  |  |  |  |
| Lysine | 2100 | 2383 | 2420 | 3428 | 2920 | 3136 | 3523 |
| Histidine | 700 | 764 | 828 | 1454 | 1088 | 1308 | 962 |
| Threonine | 1050 | 1617 | 1459 | 1537 | 1545 | 1620 | 1686 |
| Cysteine+Methionine | 1050 | 2027 | 1050 | 1491 | 1311 | 1360 | 1564 |
| Valine | 1820 | 2205 | 2073 | 1820 | 2090 | 1933 | 1820 |
| Isoleucine | 1400 | 1773 | 1708 | 1627 | 1816 | 1610 | 1593 |
| Leucine | 2730 | 2730 | 3159 | 3239 | 2730 | 2730 | 2885 |
| Phenylalanine+Tyrosine | 1750 | 3171 | 2830 | 2871 | 1960 | 2480 | 2669 |
| Tryptophan | 280 | 519 | 445 | 421 | 308 | 381 | 434 |
| **Total EAAs** | **12880** | **17189** | **15972** | **17888** | **15766** | **16559** | **17137** |

**Supplementary Table 4**: Amounts of vegetal animal foods required to provide a total amount of essential amino acids (EAAs) corresponding to the daily recommended intake of each EAA for a 70-Kg man (“C” quantity, see methods in the text for details). Such an amount is thus tailored to the individual requirements of each essential amino acid. All the reported amounts of foods are referred to edible part (See Supplementary Table 1 for further specifications).

| *RDA* |  | Soybeans 89 g | Beans 478 g | Peas 1105 g | Wheat 879 g | Maize 814 g | Rice 817 g | Potato 2856 g | Cauliflower 2169 g | Quinoa[[4]](#footnote-5) 205 g |
| --- | --- | --- | --- | --- | --- | --- | --- | --- | --- | --- |
| *Essential amino acids (mg)* | |  |  |  |  |  |  |  |  |  |
| 2100 | Lysine | 2703 | 3410 | 3846 | 2100 | 2100 | 2100 | 2630 | 2603 | 2100 |
| 700 | Histidine | 1038 | 1447 | 939 | 2003 | 2043 | 1348 | 790 | 803 | 980 |
| 1050 | Threonine | 1636 | 2044 | 3426 | 2724 | 2719 | 2010 | 1682 | 1598 | 1739 |
| 1050 | Cyst+Meth | 1050 | 1137 | 1050 | 3989 | 2499 | 2100 | 1456 | 1359 | 1157 |
| 1820 | Valine | 1930 | 2942 | 2498 | 3972 | 3842 | 3579 | 2827 | 2260 | 1970 |
| 1400 | Isoleucine | 1972 | 2655 | 2222 | 3541 | 2849 | 2500 | 1941 | 1589 | 1654 |
| 2730 | Leucine | 3273 | 4227 | 3780 | 6511 | 8367 | 4821 | 2730 | 2730 | 2867 |
| 1750 | Phe+Tyr | 3523 | 4599 | 3813 | 7513 | 6194 | 4805 | 3772 | 2797 | 3158 |
| 280 | Tryptophan^1^ | 548 | 540 | 597 | 1019 | 497 | 686 | / | / | 1488 |
| **12880** | **Total EAAs** | **17674** | **23000** | **22172** | **33372** | **31109** | **23950** | **17827** | **15738** | **17113** |

Cyst+Meth: Cysteine+Methionine. Phe+Tyr: Phenylalanine+Tyrosine. ^1^ Tryptophan concentrations in potato and cauliflowers are not reported in ref . 61. Therefore, the data were calculated with respect to the other limiting essential amino acid.

1. See ref. 10. [↑](#footnote-ref-2)
2. This weight as edible part corresponds to 3.74 eggs. [↑](#footnote-ref-3)
3. This weight as edible part corresponds to 5 eggs. [↑](#footnote-ref-4)
4. [↑](#footnote-ref-5)
